# IoT-Based Reinforcement Learning Using Probabilistic Model for Determining Extensive Exploration through Computational Intelligence for Next-Generation Techniques

**DOI:** 10.1155/2023/5113417

**Published:** 2023-10-10

**Authors:** Pradeep Kumar Tiwari, Pooja Singh, Navaneetha Krishnan Rajagopal, K. Deepa, Sampada Gulavani, Amit Verma, Yekula Prasanna Kumar

**Affiliations:** ^1^Birla Global University, Gothapatna, Bhubaneswar, Odisha, India; ^2^School of Computing Science & Engineering, Department of CSE, Galgotias University, Greater Noida, UP, India; ^3^Business Studies, The University of Technology and Applied Sciences, Salalah, Oman; ^4^Department of Computer Science and Engineering, M. Kumarasamy College of Engineering, Thalavapalayam, Karur, Tamilnadu, India; ^5^Department of MCA, Bharati Vidyapeeth (Deemed to Be University) Institute of Management, Kolhapur, Maharashtra, India; ^6^University Centre for Research and Development, Department of Computer Science and Engineering, Chandigarh University Gharuan, Mohali, Punjab, India; ^7^Department of Mining Engineering, College of Engineering and Technology, Bule Hora University, Blue Hora 144, Oromia Region, Ethiopia

## Abstract

Computing intelligence is built on several learning and optimization techniques. Incorporating cutting-edge learning techniques to balance the interaction between exploitation and exploration is therefore an inspiring field, especially when it is combined with IoT. The reinforcement learning techniques created in recent years have largely focused on incorporating deep learning technology to improve the generalization skills of the algorithm while ignoring the issue of detecting and taking full advantage of the dilemma. To increase the effectiveness of exploration, a deep reinforcement algorithm based on computational intelligence is proposed in this study, using intelligent sensors and the Bayesian approach. In addition, the technique for computing the posterior distribution of parameters in Bayesian linear regression is expanded to nonlinear models such as artificial neural networks. The Bayesian Bootstrap Deep *Q*-Network (BBDQN) algorithm is created by combining the bootstrapped DQN with the recommended computing technique. Finally, tests in two scenarios demonstrate that, when faced with severe exploration problems, BBDQN outperforms DQN and bootstrapped DQN in terms of exploration efficiency.

## 1. Introduction

IoT analytics heavily relies on reinforcement learning techniques. Making decisions consecutively is the foundation of reinforcement learning. Simply said, every next input depends on the output of the previous input and the output depends on the state of the current input. Model-based techniques may identify a solution analytically without actually engaging with the environment and can generate the precise result of every state and action interaction in RL. Because the environment is frequently too complicated to create a model, the majority of real-world problems lack models. Second, model-free solutions in RL can only monitor the behavior of the environment through real interaction with it. Machine data are often fragmented and/or include a time aspect. Also, when we accept the data coming from a certain device, gadgets can act differently depending on the context. Therefore, it is tricky to catch all instances for the data and the data cleaning phase of an algorithm. Continuous sensor data monitoring is also time-consuming and pricey. Reinforcement learning technologies embedded with IoT can help these hazards. These algorithms pick up new information on their own, freeing the programmer to focus on other important responsibilities rather than bothering about the training procedure and data collecting procedure. These developments in computational intelligence methodologies have the potential to greatly improve reliability in terms of latency and processing resources without sacrificing service quality. However, the explosive increase in the use of sensors and actuators for next-generation IoT systems generates enormous quantities of information that are processed all through the cloud, which can materially reduce processing efficiency. Sensor technology is evolving at a rate never witnessed before, driven by advances in materials' science and nanotechnology. As a result, it is becoming more accurate, smaller, and less expensive and capable of detecting things that were not imaginable before. In fact, in a few years, we will see a trillion new sensors deployed yearly since sensing technology is advancing so quickly. Actuator is a different kind of transducer that is used in many IoT systems. Simply said, an actuator works the opposite way from a sensor. It transforms an electrical input into physical movement. Actuators come in a variety of forms, such as electric motors, hydraulic systems, and pneumatic systems. In addition, IoT sensors and actuators can cooperate to provide automation on an industrial scale. Finally, over time, analysis of the data generated by these sensors and actuators might yield insightful business information. Furthermore, the traditional reinforcement learning methods may address exploration issues successfully, but there are certain constraints: the state space of a Markov decision process must be both discrete and constrained; however, in this case, it was not given sufficient attention or focus. Moreover, there are three main problems in the field of reinforcement learning [1]: (1) generalization, (2) how to balance the relationship between exploration and utilization, and (3) credit assignment problem. In recent years, the research hotspot is mainly focused on the generalization problem in large-scale state space, and there is a new research field named deep reinforcement learning [2]. The rise of this field began after the DeepMind team proposed the DQN (deep *Q*-network) algorithm [3]. The main contribution of the DQN algorithm is to combine deep learning [4] and reinforcement learning [5] and through experience playback (experience replay) and target network mechanism to solve some problems related to generalization [6]. Since then, many improved versions of the DQN algorithm have been proposed [7, 8]. These algorithms mainly focus on the generalization problem, and the main problem to be discussed in this paper is how to balance the relationship between exploration and utilization with the integration of IoT and reinforcement learning. A learning algorithm may be thought of as a stochastic mapping, or a channel in information theory, that takes training data as input and produces a hypothesis as output. The generalization error is the difference between the output hypothesis' population risk and its empirical risk on the training data. It quantifies the degree to which the learnt hypothesis suffers from overfitting. The usual method of measuring generalization error is based on either specific complexity metrics of the hypothesis space. The reciprocal data between the collection of empirical hazards of the available hypotheses and the algorithm's final output may be utilized to efficiently assess and control bias in data analysis, which is analogous to generalization error in learning issues. The total variation data between a random instance in the dataset and the output hypothesis are used to calculate the generalization error in learning problems; however, the approach is limited to bounded loss functions. Another benefit of relating the generalization error to the input-output mutual information is that, unlike the VC dimension or the uniform consistency, which only depends on the hypothesis space or the learning algorithm, the latter quantity depends on all components of the learning problem, including the dispersion of the dataset, the hypothesis space, the learning algorithm itself, and possibly the loss function. The input-output mutual information may be more closely linked to the generalization error than typical generalization-guaranteeing parameters of interest since the generalization error might substantially depend on the input dataset.

IoT analytics heavily relies on deep learning techniques. Machine data are often sparse and/or contain a temporal component. Even when we believe the data coming from a certain device, gadgets might act differently depending on the environment. Therefore, it is challenging to capture all instances for the data preprocessing/training step of an algorithm. Continuous sensor data monitoring is also time-consuming and costly. Deep learning techniques can reduce these hazards. Deep learning algorithms pick up new information on their own, freeing the developer to focus on other important tasks rather than worrying about the training process. A machine learning method called reinforcement learning involves the agent interacting with its surroundings in an effort to optimize the financial reward. The human brain communicates with the outside world and makes use of that interaction to comprehend and survive in that world. Reinforcement learning compares learning about the environment to learning about the human brain and sensory processing system. It is a process where an agent must investigate every aspect of the system to comprehend it. It is not practical in many situations due to the length of time required to converge and obtain an optimal policy. The problem with traditional RL is its dimensionality. The number of factors that an RL agent must learn increases exponentially as the environment gets more complicated. Over the network, IoT links millions of devices. Because IoT devices are so dynamic, a complicated RL may constantly acquire new data to adapt to many advanced situations. Some IoT ecosystems are so complicated that modeling them is challenging. The effort needed to simulate and solve such a complicated environment is reduced by RL. Think of a challenging IoT case where we need to develop a model to address a challenge.

The hottest supervised learning algorithms in the field of machine learning can only learn relevant patterns from data collected by humans, but what data need to be collected, or which data are important, is still up to humans to decide. However, IoT-based reinforcement learning can obtain different observation sequences by selecting different actions, and then learning-related patterns according to the obtained observation sequences. That is to say, within the scope of reinforcement learning, an algorithm is needed to decide whether to select the current optimal action (use) or choose an action (exploration) that has the potential to bring long-term benefits. However, algorithms such as DQN use a simple heuristic exploration strategy *ε*-greedy, which only randomly selects an action with the probability of *ε* (0 ≤ *ε* ≤ 1) to achieve the effect of exploration. However, such an exploration strategy is extremely inefficient. Although the DQN algorithm can reach or even exceed the human level in an Arcade Learning Environment [9], the time complexity of the algorithm is O (2N) when faced with deep exploration problems [4].

A good exploration strategy must consider the information gained brought by the selected action, and the information is mainly measured by the degree of uncertainty of the estimated value, then a good exploration strategy can be obtained only by introducing probabilistic thinking. As early as 1998, Dearden and other scholars proposed Bayesian *Q*-learning [10] to balance the relationship between exploration and utilization. Since then, other researchers have proposed reinforcement learning algorithms under the Bayesian framework [11, 12]. However, the computational processes of these algorithms are relatively complex and cannot be combined with deep learning techniques. The main problem with the combination of Bayesian reinforcement learning and deep learning is that it is intractable to obtain the posterior distribution of neural network parameters in high-dimensional state space.

The contribution of this paper is to propose a new calculation method that can generate samples of the posterior distribution of parameters, and the IoT-based deep reinforcement learning algorithms using this calculation method are collectively referred to as Bayesian deep reinforcement learning algorithms. Machine intelligence techniques govern computation, regulation, system lag, reliability, consistency, effectiveness, and energy efficiency at multiple elements in IoT ecosystems. These methodologies are in control of information gathering, linking devices to the Internet, data processing, and decision-making without interpersonal interactions. Currently, this calculation method is only applicable to value-based deep reinforcement by the combination of intelligent learning algorithms with policy-based deep reinforcement learning algorithms. Combining the bootstrapped DQN with this calculation method, a new algorithm BBDQN (Bayesian bootstrapped DQN) is obtained, and the exploration efficiency of the algorithm is verified through experiments in two environments, one of which is a lattice world with a deep exploration structure. Another experimental environment is the classic control problem Mountain Car, which does not have a deep exploration structure itself, slightly modify its reward function to make it have a deep exploration structure, and then conduct experiments on the modified Mountain Car. The experimental results show that BBDQN can solve the deep exploration problem, and its exploration efficiency is better than the DQN algorithm using a random exploration strategy (i.e., *ε*-greedy) and the bootstrapped DQN algorithm. Through the use of randomized value functions, the straightforward algorithm bootstrapped DQN explores in a computational and statistically efficient way. Bootstrapped DQN engages in temporally extended (or deep) exploration as opposed to dithering tactics such as epsilon-greedy exploration; this can result in tenfold quicker learning. We illustrate these advantages in the expansive arcade learning environment and complicated stochastic MDPs. Bootstrapped DQN significantly reduces learning times and enhances performance in the majority of Atari games. In a traditional bootstrap, you simulate sampling your data from a population by collecting samples with substitutions from your data. This procedure is repeated *K* times to mimic drawing your sample several times, allowing you to assess the potential variability estimate of your statistic (a function) over various samples drawn from the same population (*Xk*). Consequently, we are emulating the statistic's “sampling distribution” (variability due to the sampling process). In the specified Bayesian bootstrap, you are estimating the posterior distribution of the estimates of the statistic *ϕ* (*X*) and the distribution of your data *X* = {*x*1, *x*2,…, *xN*}. It is a nonparametric model in which your data points are assumed to have a categorical distribution (the likelihood).

Even though the DQN has already considerable success, it may still be enhanced with the aid of a variety of different methods, including a more precise approximation approach for the state action-value function and prioritized experience replay. Due to its capacity to balance exploration and exploitation in reinforcement learning, the exploration approach should be the most crucial of these strategies. The exploration strategy determines the course of action to pursue given the current situation and has the potential to affect how the process will ultimately develop in the future. Given its significance, several exploration tactics have been suggested. The strategy employed in the first DQNs and possibly the one that is utilized the most is the epsilon-greedy one. The greedy approach mostly selects the action that is thought to be the best, but it also randomly selects an action with little probability. An activity that has been proven to be a poor option may be repeatedly picked in the succeeding decision-making process since the activities are chosen entirely at random for investigation. To increase the effectiveness of exploration, add some Gaussian noise. For jobs with well-defined incentives, these heuristic strategies are enough.

A significant issue for reinforcement learning is still effective exploration in complicated situations. We suggest the bootstrapped DQN, a straightforward technique that uses randomized value functions to explore in a way that is both computationally and statistically efficient. The bootstrapped DQN engages in temporally extended (or deep) exploration as opposed to dithering tactics such as epsilon-greedy exploration; this can result in tenfold quicker learning. We illustrate these advantages in the expansive Arcade Learning Environment and complicated stochastic MDPs. The bootstrapped DQN significantly reduces learning times and enhances performance in the majority of Atari games. The bootstrapped DQN builds many posterior estimates using the same original data. The random initialization of the weights for the heads is what causes the variety of approximate solutions. This implies that these heads first try random actions (due to varied random initials) but that some (but not all) of the heads will learn from it due to bootstrapping when some heads discover a favorable state and generalize to it. Other heads will eventually either discover additional excellent states or come to understand the greatest good states discovered by the other heads. Simply employing the bootstrapped DQN as an exploitative approach is quite good in and of itself, even better than the vanilla DQN. This is due to bootstrapped DQN's deep exploration capabilities, since it may employ the best states, it knows while simultaneously planning to try out states it does not know anything about. Even in the films, it is clear that the heads agree on all critical decisions but disagree on other, less significant actions.

## 2. Related Work

The exploration efficiency of the reinforcement learning algorithm directly affects the sample efficiency of the algorithm, and improving the exploration efficiency can reduce the number of time steps required to train the agent. The Bayes policy is a criterion for measuring the exploration efficiency of reinforcement learning algorithms; however, computation of Bayesian optimal policies is tricky because the computation time grows exponentially with the problem space (states and actions) [13]. There are many studies considering exploration efficiency, and Liu et al. [14] were the authors to propose it, and their work confirmed that polynomial-time reinforcement learning algorithms must use multiperiod exploration. Based on their work, a series of tabular reinforcement learning algorithms has been proposed [15–21]. The exploration methods mentioned in these papers are more efficient than *ε*-greedy and Boltzmann exploration, but these methods all cannot handle the curse of dimensionality. Deep reinforcement learning algorithms [3, 22, 23] are proposed in recent years to deal with large state spaces tend to use *ε*-greedy exploration. These algorithms can get good results in Atari arcade games and Go, but they do not have any practical applications; the inefficient exploration efficiency makes these algorithms only trained in simulated environments. By randomly selecting between exploration and exploitation, epsilon-greedy is a straightforward strategy for balancing exploration and exploitation. Epsilon refers to the likelihood of deciding to explore, and the epsilon-greedy, with a low probability of exploring, exploits most of the time. Theoretical computer science, optimization, machine learning, and decision theory all use exponential weighting schemes as basic tools. Exponential weighting systems, often known as Boltzmann, Gibbs, or softmax exploration strategies, are frequently employed in reinforcement learning to balance exploration and exploitation. The chance of selecting an arm in the most popular variant of Boltzmann exploration is inversely related to an exponential function of the empirical mean of the reward for that arm.

For the exploration of large-scale state space, some researchers proposed a model learning algorithm [24]. The problem with this method is that it can only solve simple model problems. For complex models, the calculation of this method is also difficult. Some researchers have proposed a policy learning algorithm [25], which mainly solves the problem of continuous action space, such as robot control, but when the size of the policy space is exponential, this method cannot guarantee the efficiency of exploration. Another class of approaches encourages exploration by assigning rewards to infrequently visited states based on pseudocounts [26] or density models [27].

How to ensure the exploration efficiency, and generalization of the algorithm has always been a difficult problem in reinforcement learning, and some researchers have thought of using Bayesian thinking to deal with this problem, such as the Bayesian *Q*-learning algorithm [10], but this algorithm can only deal with state A limited number of problems; there is also the RLSVI (randomized least-square value iteration) algorithm proposed in this literature [28], which is only suitable for linear function approximates and cannot be combined with neural networks, a nonlinear function approximate; while the algorithm proposed in this literature [29] uses the neural network as the feature extractor that takes the input of the last layer of the network as a feature and then uses Bayesian linear regression to calculate the *Q*-value. RLSVI works similarly to least-squares value iteration (LSVI) and has many of the same principles as other closely related algorithms such as TD, LSTD, and SARSA. The difference between RLSVI and the alternatives is that the algorithm explores by randomly sampling statistically plausible value functions, whereas the alternatives are typically used in conjunction with action-dithering schemes such as Boltzmann or epsilon-greedy exploration, which results in highly inefficient learning. The randomized least-squares value iteration (RLSVI) technique rapidly investigates and generalizes value functions with linearly parameterized values. It is, however, reliant on hand-designed state representation, which necessitates engineering effort for each scenario.

Research in Bayesian neural networks has not been mainstreaming [30, 31]; however, there have been recent signs of a resurgence, with many concerns about quantifying the uncertainty of datasets. Methods [32] have been proposed, and the authors of this paper are inspired by these methods and propose a deep reinforcement learning algorithm that can efficiently compute Bayesian posteriors. Because they provide us with whole distribution across the regression parameters, Bayesian regression methods are known to be particularly powerful. Bayesian linear regression offers a built-in technique for calculating insufficient or unevenly distributed data. The coefficients can have a prior applied to them so that, in the absence of data, the prior can be used instead. In Bayesian linear regression, statistical analysis is carried out under Bayesian interface conditions. To create linear regression, we do not utilize point estimates but rather probability distributions. An output is produced using a probability distribution rather than more traditional methods of regression. Bayesian linear regression seeks to identify posterior rather than model parameters.

## 3. Background Knowledge

A collection of potential world states *S*, a set of models, a set of potential actions *A*, a real-valued reward function *R* (*s*, *a*), and a policy are all components of a Markov Decision Process (MDP) model. Each possible state for the agent is represented by a set of tokens called a state. A model, often known as a transition model, describes how an action changes a state. Specifically, the transition *T* (*S*, *a*, *S*′) describes a situation in which being in state *S* and performing an action “*a*” transports us to state *S*′ (*S* and *S*′ may be the same). All conceivable actions are included in an action *A*. The collection of actions that can be executed while in state *S* is defined as *A* (*s*). A reward is a reward function with a real value. The reward for merely existing in the state *S* is denoted by *R* (*s*). The reward for existing in a state *S* and doing an action “*a*” is indicated by the expression *R* (*S*, *a*). The reward for being in a state *S*, doing an action “*a*,” and then ending up in a state *S*′ is denoted by the formula *R* (*S*, *a*, *S*′). The Markov Decision Process has a solution in the form of a policy. A mapping from *S* to *a* is a policy. It signifies that when in state *S*, action “*a*” should be conducted. Reinforcement learning problems are usually modeled as Markov Decision Processes. The Markov Decision Process used in this paper can be expressed as (*S*, *A*, *T*, *R*, *γ*), where *S* is the state space, *A* is the action space, and *A*(*s*), *s* ∈ *S* is the action set available in state *s*; T is the state transition probability, *T*(*S*_*t*+1_|*s*_*t*_, *a*_*t*_) represents the probability of transitioning to state *S*_*t*+1_ after taking action at state *s*_*t*_; *R* is reward functioned, *R*_*s*,*a*_ denotes the mean value of the reward distribution obtained after taking action *a* in state *s*, and let *r* denote the immediate reward obtained after taking action *a* in state *s* (it can also be understood as a sample obtained from the reward distribution), and then there is *R*_*s*,*a*_=Ε[*r* |*s*_*t*_=*s*, *a*_*t*_=*a*];  *γ* ∈ [0,1] as the discount parameter used to control the weight of future rewards.

The goal of reinforcement learning is to learn a policy that maximizes the discounted cumulative reward obtained by the agent. Let *π* denote the policy and *π*(*a*|*s*) denote the probability of taking action *a* in state *s*. Then, introduce the concept of action-value function (*Q* function), the action-value function *Q*_*π*_(*s*, *a*) table under the policy *π*, and the agent can obtain the expected discounted cumulative reward after taking action states, namely,

(1) $$\begin{array}{c} Q_{\pi }\left(s,a\right)=E_{\pi }\left[\overset{\infty }{\underset{t=0}{\sum }}\gamma^{t}r_{t}\left|s_{0}=s,a_{0}=a\right.\right]\text{.} \end{array}$$

This equation can also be expressed recursively:

(2) $$\begin{array}{c} Q_{\pi }\left(s,a\right)=R_{s,a}+\gamma \underset{s^{t},a^{t}}{\sum }\left(s^{\prime }\left|s,a\right.\right)\pi \left(a^{\prime }\left|s^{\prime }\right.\right)Q_{\pi }\left(s^{\prime },a^{\prime }\right)\text{.} \end{array}$$

We denote the optimal policy by *π*^*∗*^ and the optimal action-value function by *Q*^*∗*^. Since the optimal policy is the policy that obtains the maximum discounted cumulative reward, the *Q*^*∗*^ function can be written as follows:

(3) $$\begin{array}{c} Q^{*}\left(s,a\right)=R_{s,a}+\underset{s^{t}}{\sum }T\left(s^{\prime }\left|s,a\right.\right)\max_{a^{\prime }}\;Q^{*}\left(s^{\prime },a^{\prime }\right)\text{.} \end{array}$$

Equation (1) is also known as the Bellman optimality equation. For each pair of *s*, *a*, there is an equation. By combining all these equations, a system of equations is obtained, which can be solved by linear programming methods. This system of equations yields the optimal action-value function *Q∗*(*s*, *a*) for all *s*, *a*. The optimal action-value function can also be solved by dynamic programming methods such as policy iteration or value iteration. The optimal strategy can be expressed by the optimal action-value, that is, for all states *s*, select the action *a*=argmax *a* *Q∗*(*s*, *a*) that maximizes the optimal action-value function in this state; if there are multiple actions satisfying with this condition, one of these actions can be randomly selected. It can be seen from equation (1) that this method requires a known environment model—state transition probability *T* and reward the function *R* to be calculated. If the environment model is not known, other methods are needed to find the maximum value. Optimal strategies, such as *Q*-learning algorithms, are used to calculate the optimal action-value function, iteratively, and the update rule is

(4) $$\begin{array}{c} Q\left(s,a\right)\leftarrow Q\left(s,a\right)+\alpha \left[r+\gamma \max\;Q\left(s^{\prime },a^{\prime }\right)-Q\left(s,a\right)\right]\text{.} \end{array}$$

Among them, *α* is the learning rate, and the reward *r* and the next state, *s* are both fed back to the agent by the environment. In the case of limited state space and action space, as long as the learning rate sequence satisfies the random approximation condition and all state-action pairs are in the case of continuous updates, the algorithm converges to the optimal value function *Q*^*∗*^ [5] with probability 1.

In large-scale state spaces, the traditional reinforcement learning algorithm *Q*-learning is no longer applicable, but this problem can be solved by combining function approximation techniques. The combination of neural networks and reinforcement learning is a hot research topic in recent years, and the DQN algorithm is a model in this regard. When using a neural network, the action-value function can be expressed as *Q*_*θ*_(*s*, *a*), where *θ* is a parameter of the neural network. We compute the target value from feedback from interacting with the environment:

(5) $$\begin{array}{c} \gamma =r+\gamma \;\max Q_{\theta }\left(s^{\prime },a^{\prime }\right)\text{.} \end{array}$$

With the target value and the predicted value, the parameter *θ* of the neural network can be updated by the stochastic gradient descent algorithm. The update rule is

(6) $$\begin{array}{c} \theta \leftarrow \theta -\alpha \nabla_{\theta }{\left[y-Q_{\theta }\left(s,a\right)\right]}^{2}\text{.} \end{array}$$

If you do this directly, the effect is not very good because the neural network is a supervised learning method, which requires that each training sample is independent of each other, and the samples collected by reinforcement learning are related to each other, and each update of the parameters will affect the target value, causing the target value to be unstable. As mentioned in the DQN algorithm, only two mechanisms are needed to alleviate this problem: experience replay and the target network. The experience replay mechanism is that, each time the agent interacts with the environment, the information (*s*, *a*, *r*, *s*′) of the interaction is stored in the memory pool (replay buffer), and the agent randomly selects from the memory pool for each update. We take a certain number of samples and use these samples to update the parameters. The target network mechanism is that the agent maintains two *Q* networks, one network is used to select actions and update them in real time and the other network (target network) is used to calculate the target value and will not update in real-time the target value used in each update, and it can be expressed as

(7) $$\begin{array}{c} \gamma =r+\gamma \;\max\;Q^{target}\left(s^{\prime },a^{\prime }\right)\text{.} \end{array}$$

Among them, *Q*^target^ represents the target network, and only after the specified time step has passed, the parameters of the two networks will be synchronized once.

## 4. In-Depth Exploration of the Problem

The chain problem mentioned is a kind of deep exploration problem, and this paper will use this problem to analyze the exploration efficiency of the *ε*-greedy strategy and Boltzmann exploration. To simplify the calculation, some modifications have been made to the chain problem as discussed in the literature [10]. *ε*-greedy exploration is to randomly choose one of all available actions with probability *ε*, and choose the best action for the moment with probability 1 − *ε*. The probability of selecting each action in Boltzmann's exploration is proportional to the estimate of the action-value function, which is calculated as

(8) $$\begin{array}{c} \pi \left(a\left|s\right.\right)=\frac{e^{Q`\left(s,a\right)/t}}{\sum_{i}e^{Q`\left(s,a\right)/t}}\text{.} \end{array}$$

**Figure 1:**
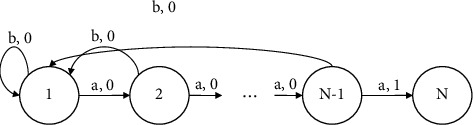
Chain problem.

Among them, *Q͂* is the estimation of the action-value function, *t* is the temperature parameter, the larger the value of *t* is, the closer the strategy is to the random strategy, and the smaller the value of *t* is, the closer it is to the greedy strategy. A chain problem is used to analyze the exploration efficiency of *ε*-greedy exploration and Boltzmann exploration. The state transition diagram of this problem is shown in Figure 1. Each arrow in the diagram corresponds to an action and reward. The starting state of the problem is 1, and the termination state is *N*. Suppose the length of each episode (episode) *H*=*N* − 1, and no matter in which state, the choice of action *a* will have, a probability of 0.2 failure (failure means that the selected action and the actual action are inconsistent) and action *b* is deterministic. The optimal strategy for this problem is to always choose action *a*, and the average reward that the optimal strategy can get in each cycle is the probability (1 − 0.2)^*N*−1^ that the strategy successfully reaches *N*. Whether it is *ε*-greedy or Boltzmann exploration, since there is no reward before reaching the target state *N* (i.e., the reward is 0), the estimated values of action *a* and action *b* are equal in all states, and the action selection is completely random; in this case, the probability of reaching state *N* through exploration is (1 − 0.2)^*N*−1^ × 2^−(*N* − 1)^, just take the reciprocal of this probability to get the average required amount of reaching state *N* through exploration. The number of cycles, with *l* representing the required number of cycles, the following relationship is established:

(9) $$\begin{array}{c} the\;E\left[l\right]=\frac{1}{{\left(1-0.2\right)}^{n-1}}2^{N-1}>2^{N-1}\text{.} \end{array}$$

From this inequality, it can be determined that a reinforcement learning algorithm using an *ε*-greedy strategy or Boltzmann exploration has a time complexity of *O*(2^*N*^) when faced with a deep exploration problem such as the chain problem, where *N* represents the size of the problem.

The exploration efficiency of epsilon-greedy exploration and Boltzmann exploration is examined using a chain problem. Figure 1 depicts the state transition diagram for this problem. Each arrow in the graphics represents an activity and its associated reward. The problem's initial state is 1 and its final state is *N*. The described chain issue is a type of deep exploration problem, and this study will utilize it to compare the exploration efficiency of the epsilon-greedy method with Boltzmann exploration. Epsilon-greedy exploration is the process of randomly selecting one of all potential actions with probability, and then selecting the optimal action for the moment with probability. In Boltzmann's exploration, the likelihood of picking each action is proportional to the estimated action-value function.

## 5. Bayesian Deep Reinforcement Learning

The benefits of exploration can be estimated by the value of information. The so-called value of information refers to the degree to which the information obtained through exploration leads to the improvement of the quality of future decision-making, and how quantifying the information obtained through exploration is the key. According to information theory, the amount of information brought by the exploration can be calculated by the uncertainty of the estimated value of the action selected by the exploration. The quantitative treatment of the ideas, parameters, and regulations regulating message transmission through communication networks is known as information theory. It was established by Claude Shannon in the middle of the 20th century and has since developed into a vibrant branch at the intersection of communication theory and mathematics, promoting the advancement of other scientific disciplines. Some people consider information theory to be a subset of probability theory since the methods utilized in it are probabilistic in nature. Whether an analog or digital communication technology is used, information is its source. Information theory is a mathematical method for studying information coding, as well as quantification, storage, and communication. In [10], a Bayesian method is proposed to maintain this information; however, in this paper, the number of state-action pairs of the problem discussed is limited, and all the obtained state-action pairs can be recorded by recording all the variance and then calculate the amount of information. When the number of state-action pairs is too large, reinforcement learning will consider combining generalization techniques to solve this problem. If a linear function approximator is used, it can be estimated according to the Bayesian linear regression mentioned in [33] value variance. However, in recent years, research related to deep learning has shown that the strong generalization ability of neural networks is much stronger than that of linear function approximators. Therefore, the combination of deep learning and reinforcement learning technology has become a major trend, and a new field of research is called deep reinforcement learning. Due to the complex structure of the neural network and the huge number of parameters, the variance of the estimate cannot be calculated by the calculation method mentioned in the literature [33], so this paper proposes a new calculation method. This method obtains a sample of the posterior distribution of the neural network parameters by calculation at each time step. Since the estimated variance of different actions is different, the action with high variance has a larger amount of information, and its sampling value may also be larger. Therefore, the probability of being selected is also higher. For example, there are two actions whose *Q*-value obeys a Gaussian distribution. Since action 1 is selected many times, its variance is smaller than that of action 2. Suppose that the variance of action 1 is 1, and the variance of action 2 is 10. In addition, the variance of action 1 is 1. Action 1 has a mean historical return of 3 and action 2 has a mean historical return of 1, so action 1 follows a Gaussian distribution with mean 3 and variance 1, while action 2 follows a Gaussian distribution with mean 1 and variance 10, resulting in action 2. Due to the large variance, the probability that its sampled value is greater than that of action 1 is also greater. The following sections describe in detail how to apply Bayesian methods in deep reinforcement learning algorithms.

The input to a neural network can be a one-dimensional array, a two-dimensional matrix, or even a three-dimensional image. For the sake of clarity, *x* ∈ *ℜ*^*d*^ is used to represent the state feature, that is, the input value of the neural network, plus the target value *y* to form the training set, which is expressed as *D*={(*x*_*i*_*y*_*i*_)}_*i*=1_^*n*^. The posterior distribution of the linear regression model *f*_*θ*_ (*x*_*i*_)=*θ*^*T*^*x*_*i*_ parameters is calculated and later extended to the case of neural networks. The model parameters are expressed as *s* ∈*ℜ*^*d*^, and the prior distribution of the parameters *θ* is assumed to be *N*(*θˉ*, *λI*), and the observed target value has a certain noise, that is, *y*_*i*_=*θ*^*T*^*x*_*i*_+*w*_*i*_, where *w*_*i*_ ∝ *N*(0, *σ*^2^). For noise, the noise of each sample is independent of each other. According to Bayes' theorem, the posterior distribution of the parameter *θ* can be expressed as

(10) $$\begin{array}{c} p\left(\theta \left|D\right.\right)=\frac{p\left(D\left|\theta \right.\right)p\left(\theta \right)}{p\left(D\right)}\text{.} \end{array}$$

In Bayesian theory, *p*(*D*|*θ*) is called the likelihood, *p*(*θ*) is called the prior, and *p*(*D*) is called the evidence. According to the derivation of the literature [33], the posterior distribution of the parameter *θ* obeys the multivariate Gaussian distribution with the mean (12) and the covariance (13):

(11) $$\begin{array}{c} E\left[\theta \left|D\right.\right]={\left(\frac{1}{\sigma^{2}}X^{T}X+\frac{1}{\lambda }I\right)}^{-1}\text{,} \end{array}$$

(12) $$\begin{array}{c} cov\left[\theta \left|D\right.\right]={\left(\frac{1}{\sigma^{2}}X^{T}X+\frac{1}{\lambda }I\right)}^{-1}\text{.} \end{array}$$

Among them, *X* ∈ *ℜ*^*n*×*d*^ is the matrix obtained by splicing *n* sample inputs *x*_*i*_*i* and *y* ∈ *ℜ*^*n*^ is the vector obtained by splicing *n* target values *y*_*i*_. This posterior distribution can be inferred by methods such as Markov Chain Monte Carlo, but to extend to nonlinear model artificial neural networks, the posterior distribution is expressed in another form:

(13) $$\begin{array}{c} \theta^{\prime }\leftarrow \;{\left(\frac{1}{\sigma^{2}}X^{T}X+\frac{1}{\lambda }I\right)}^{-1}\left(\frac{1}{\sigma^{2}}X^{T}\left(y+\delta \right)+\frac{1}{\lambda }\theta^{\prime }\right)\text{.} \end{array}$$

Among them, *δ* ∈ *ℜ*^*n*^ is a random vector, each component *δ*_*i*_ comes from the Gaussian distribution (0, *σ*^2^), and the sampling of each component is independent of each other, and *θ̂* comes from the parameter prior distribution *N*(*θ*', *λI*). It can be shown that *θ͂*' and *θ* are equivalent because

(14) $$\begin{array}{c} E\left[\theta^{\prime }\left|D\right.\right]={\left(\frac{1}{\sigma^{2}}X^{T}X+\frac{1}{\lambda }I\right)}^{-1}.\;\left(\frac{1}{\sigma^{2}}X^{T}\left(y+E\left[\delta \left|D\right.\right]\right)+\frac{1}{\lambda }E\left[\theta^{\prime }\left|D\right.\right]\right)=E\left[\theta \left|D\right.\right]\text{.} \end{array}$$

That is, the means of *θ͂*' and the mean of *θ* are equal, and there are

(15) $$\begin{array}{c} cov\left[\theta \left|D\right.\right]={\left(\frac{1}{\sigma^{2}}X^{T}X+\frac{1}{\lambda }I\right)}^{-1}.\left(\frac{1}{\sigma^{4}}X^{T}E\left[{\delta \delta }^{T}\left|D\right.\right]X+\frac{1}{\lambda^{2}}E\left[{\theta^{\prime }\theta^{\prime }}^{T}\left|D\right.\right]\right)\text{ .} \end{array}$$

((1/*σ*^2^)*X*^*T*^*X*+(1/*λ*)*I*)^−1^=cov[*θ*|*D*] and ΕδδTD=σ2 I,Εθ^'θ^'TD=λI, that is, the covariance of *θ͂*' is equal to the covariance of *θ*. With *θ*', the posterior parameters can be directly calculated, which is the key to combine Bayesian methods and neural networks. Calculating *θ*' requires adding noise to the target value, which can be obtained by open delta number generation noise sample open *δ*_*i*_ for each sample (*x*_*i*_, *y*_*i*_). In addition, the reinforcement learning algorithm will continuously collect new data and modify its model parameters over time. Therefore, formula (15) is rewritten into an iterative calculation method to adapt it to the form of online learning. The update rules are as follows:

(16) $$\begin{array}{c} \theta^{\prime }\leftarrow \theta^{\prime }-\nabla_{\theta }L\left(f_{\theta }+f_{\theta };D_{noise}\right)\text{.} \end{array}$$

Among them, ∇_*θ*_*L*(*f*_*θ*_ + *f*_*θ*_; *D*_noise_) is the loss function and *f*_*θ*′_ + *f*_*θ*_ indicates that the output of the prediction function is obtained by adding the outputs of two neural networks, one of which has the posterior parameter *θ͂*' and the other network has the prior. The parameter *θ*' ;  *D*_noise_ represents the dataset with added noise, i.e., *D*_noise_ = {(*x*_*i*_*y*_*i*_ + *δ*_*i*_)}_*i*=1_^*n*^. Equation (16) is to update the posterior parameter *θ͂*, and the prior parameter *θ̂* will not change after it is determined in the initialization stage. We combine formula (16) for calculating the posterior parameters and the bootstrapped DQN method, since the DQN method adopts the experience replay mechanism, and the way of sampling samples from the memory pool in the experience replay mechanism is equivalent to inject Gaussian noise into the data [34], so there is no need to explicitly add noise; the dataset is represented by *D*_sample_ = {(*x*_*i*_*y*_*i*_ + *δ*_*i*_)}_*i*=1_^*n*^, where *n* represents the number of samples drawn from the memory pool at each update, also known as the batch size. We denote the target network as *f*_*θ*′_, and then the loss function can be written as

(17) $$\begin{array}{c} opensL\left(f_{\theta^{\prime }}+f_{\theta^{\prime }};D_{sample}\right)≔\underset{t\epsilon D_{sample}}{\sum }\left(y_{i}-\left(f_{\theta^{\prime }}+f_{\theta^{\prime }}\right)\left(x_{i}\right)\right)\text{.} \end{array}$$

The method that combines the Bayesian method and bootstrapped DQN is called the BBDQN algorithm, see Algorithm1 for an overall description of the algorithm.

### 5.1. BBDQN Algorithm

The Bayesian bootstrap, like other bootstrapping approaches, can enhance probabilistic forecasts by employing preprocessed data with replacement, which allows for distinguishing the output predictions. The Bayesian bootstrap in this research works analytically to obtain the posterior distributions of the QR model parameters, distinguishing itself from the classic bootstrap, which depends on random choices from the available input data. Because the observations are treated as fixed in Bayesian inference, this new perspective appears to be compatible with the Bayesian method. Indeed, the determination of the mean using the Bayesian bootstrap varies only in the weight distribution. However, from a conceptual viewpoint, the Bayesian bootstrap differs significantly from the frequentist form.

## 6. Experimental Analysis

### 6.1. Lattice World

**Figure 2:**
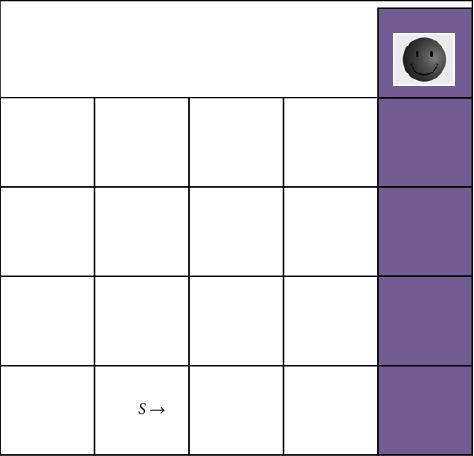
Grid world with deep exploration structure.

First, test the exploration efficiency of the BBDQN algorithm in the grid world shown in Figure 2. The white grid represents the scale of the grid world, and the gray grid is the terminal state. Due to space constraints, the grid world in Figure 2 has a scale of only 4 × 4, while the scale of the lattice world used in the experiment is 20 × 20, but this does not prevent the use of the small-scale lattice world of Figure 2 to describe its dynamic model. In Figure 2, the state *S* is the initial state, and the agent has always maintained a speed of moving to the right +1. The available actions in each state are up and down. If you choose up, then the next step will reach the upper right state of the current state, and if down is selected, the next step will reach the lower right state of the current state. If the agent is at the bottom of the grid world, the downward action can be understood as walking against the wall. At this time, the next state will be to the right of the current state. If you select the action up in the upper right corner of the white grid, you can reach the top of the gray grid. To get a reward of +1, there are no rewards in other states, so you must always choose the action up to get the reward, but the action up has a cost, and the cost is related to the size of the grid world. Assuming that the size of the grid world is *N* × *N*, each selection action up will bring a reward of −0.01/*N*, while choosing action down has no cost, the reward is 0. In fact, this problem is a two-dimensional extended version of the chain problem mentioned in Paper 4. The input can be represented as a one-hot matrix *x*_*i*_ ∈ {0,1}^*N*×*N*^, the position of the agent in the matrix is 1, and the other positions are all 0s. In this experiment, compared with the DQN using *ε*-greedy strategy and bootstrapped DQN, the hyperparameters used by BBDQN algorithm are shown in Table 1, where 0 ∈ *ℜ*^*d*^ represents a vector with all 0 components.

In the grid world, simple is the norm. The left-bottom corner of your agent or robot serves as the “start” point, and it terminates at either +1 or −1, which corresponds to the associated reward. The agent has four alternative movements at each step, including up, down, left, and right, while the black block is a wall that your agent cannot pass through. Our first solution assumes that each action is deterministic, i.e., the agent will move in the direction it desires to mov in order to simplify things. The wall, on the other hand, will stay in place if the agents run into it. This is where artificial intelligence comes into play, because our bot should be able to learn from the procedure and think like a person. Value iteration is the key to the magic. Once our agent discovers a path to reward +1, should it stick to it and always take that line (exploitation), or should it give other paths a shot (exploration) and expect a shorter path? In reality, we will balance exploitation and exploration in order to keep our agent from being stuck in the local optimum. Our agent will pick an action depending on the exploration rate.

**Figure 3:**
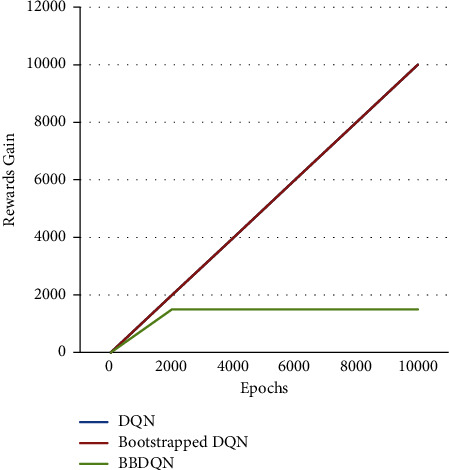
Algorithm performance over epochs for rewards gain.

The experimental results are shown in Figure 3, where the algorithm performance is measured with regret. Regret refers to the difference between the maximum cumulative reward and the actual cumulative reward. Because DQN has never found a rewarded area in the grid world, its cumulative reward is less than 0, and its regret is constantly growing. As can be seen from Figure 3, the BBDQN can learn the optimal policy in less than 2000 epochs, while the DQN does not learn a good policy after 10000 epochs. In fact, after 1 million cycles of experiments, the DQN still did not converge.

The time complexity of the DQN algorithm using the *ε*-greedy strategy is O (2*N*) when faced with deep exploration problems. This paper also tests the performance of the bootstrapped DQN algorithm. The action selection of this algorithm is not realized by *ε*-greedy, and the algorithm improves its exploration efficiency by adding ensemble technology on the basis of the DQN [10], but it cannot be used in solving this deep exploration problem in the short term. The regret of the bootstrapped DQN and DQN over 10 000 cycles has only a small difference, which is not visible in Figure 3 and must be zoomed in to see the difference. Specifically, the DQN has accumulated regrets of 9 over 10000 cycles.

Figure 3 is the performance curve of the algorithm when the grid world size is 20 × 20. It is possible to assume that the size of the lattice world is *N* × *N* and analyze the relationship between algorithm exploration efficiency and *N*. As in Paper 4, the number of cycles required for the algorithm to discover the reward for the first time is used as a measure of exploration efficiency, and 6 data samples are obtained through experiments, which are shown in Table 2, where *l* represents the first discovery of the reward by the algorithm. In order to express the relationship between the two, polynomial regression is used to fit these 6 points, which is expressed as follows:

(18) $$\begin{array}{c} l=b+w_{1}N+w_{2}N^{2}+\ldots +w_{m}N^{m}\text{.} \end{array}$$

Among them, *w*_1_, *w*_2_,…, *w*_*m*_*m* are the parameters of the *m* degree polynomial, which can be obtained by the least-square method. We tested multiple *m* values and found that when *m* is 3, the mean square error is the smallest, that is, the relationship between the two can be approximated by a 3rd degree polynomial as follows:

(19) $$\begin{array}{c} l\approx -2.76+9.30N-6.84N^{2}+1.63N^{3}\text{.} \end{array}$$

Among them, the parameter retains two decimal places. Therefore, when the BBDQN algorithm faces deep exploration problems, the time complexity of the algorithm is at the polynomial level, specifically *O*(*N*^3^), which is better than the random exploration strategy *ε*-greedy time complexity of *O*(2^*N*^).

### 6.2. Further Analysis on Lattice World

In Figure 3, there is no obvious difference between the DQN algorithm and the bootstrapped DQN algorithm, but this does not mean that the exploration efficiency of the two algorithms is the same. In fact, the bootstrapped DQN algorithm is more efficient than the DQN. To prove this conclusion, a series of experiments were carried out.

**Figure 4:**
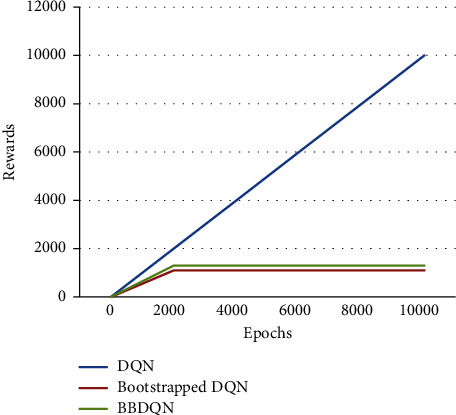
Algorithm performance over epochs for rewards gain (*N* = 10 and *K* = 10).

**Figure 5:**
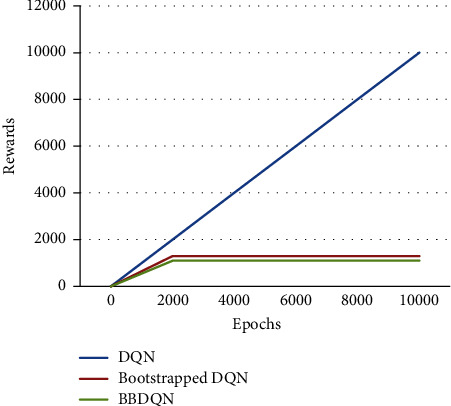
Algorithm performance over epochs for rewards gain (*N* = 20 and *K* = 20).

In the previous section, it was mentioned that the size of the lattice world is represented by *N* × *N*, and the number of learners is represented by *K*. The performance of each algorithm is compared when *K* is fixed at 10 and *N* is 10, 20, and 30. In addition, the performance of each algorithm is compared when *N* is fixed at 20 and *K* is 10, 20, and 30. When the lattice size of the world becomes smaller, the superiority of the bootstrapped DQN over the DQN is reflected. As shown in Figure 4, when *K* is 10 and *N* is 10, the regret of the DQN algorithm keeps growing, while the bootstrapped DQN and BBDQN find the optimal policy within 1000 cycles. In addition, when the number of learners increases, the superiority of the bootstrapped DQN over the DQN can also be reflected. As shown in Figure 5, when *K* is 30 and *N* is 20, the bootstrapped DQN algorithm finds the optimal policy at around 2000 cycles.

The results of all experiments are shown in Table 3, where the bootstrapped DQN is denoted as the BDQN due to space constraints. In addition, the experimental results in Section 6.1 are the results shown in the table with *K* = 10 and *N* = 20. In Table 3, the algorithm can learn the optimal policy in 10,000 epochs.

Regrettably shown in bold, it can be seen that the DQN fails to find the optimal policy within 10,000 epochs in all experimental settings, while the bootstrapped DQN only works when *K* = 10, *N* = 10 (the size of the lattice world decreases), or *K* = 30 and *N* = 20 (the number of learners increases); the optimal policy can be learned in 10,000 epochs, while the BBDQN can learn the optimal policy in 10,000 epochs under all settings. It can also be seen from Table 3 that when the size of the lattice world is small (*N* = 10), the performance of the BBDQN algorithm and the bootstrapped DQN algorithm is not much different because the BBDQN adds a random initialization first.

The performance of the BBDQN is even slightly lower than the bootstrapped DQN, but when the scale of the grid world increases, the advantages of the BBDQN algorithm proposed in this paper become more obvious, which is also the embodiment of the BBDQN more suitable for solving deep exploration problems; in addition, BBDQN algorithm performance of the method does not increase with the number of learners, which means that the space requirement of the BBDQN is lower than that of the bootstrapped DQN because the more the number of learners, the more space is required to store each learned parameter, and these algorithms all use neural networks as function approximators, and the parameters of each network are in millions, and the BBDQN does not need to increase the number of learners like the bootstrapped DQN to improve algorithm performance (as shown in Table 3), so the space requirement of the BBDQN is lower than the bootstrapped DQN.

## 7. Conclusion

A variety of learning and optimization methods form the foundation of computational intelligence. Therefore, incorporating cutting-edge learning methods to balance the relationship between exploration and exploitation is an inspiring area, particularly when merging it with IOT. As a result, IoT collected the scattered data and screened the information out of it using computational algorithms, and reinforcement learning performed the deep exploration with the ultimate emphasis. While disregarding the issue of identifying and taking advantage of the dilemma, the reinforcement learning approaches developed in recent years have mostly concentrated on integrating deep learning technology to enhance the algorithm's generalization capabilities. In order to increase the effectiveness of exploration, a deep reinforcement algorithm based on computational intelligence is proposed in this study, using intelligent sensors and the Bayesian approach. In addition, the technique for computing the posterior distribution of parameters in Bayesian linear regression is expanded to nonlinear models such as artificial neural networks; also, this calculation method is combined with the bootstrapped DQN to obtain the BBDQN algorithm. The result of the experiments in two environments proves that the exploration efficiency of the BBDQN algorithm is better than the DQN algorithm using the *ε*-greedy strategy and the bootstrapped DQN algorithm.

The direction of further research is when the method of calculating the posterior distribution of the *Q* function parameter in this paper is used to calculate the posterior distribution of the policy function *π*_*θ*_ parameter in the policy gradient method, the policy gradient method can effectively improve the face of the policy gradient method and exploration efficiency when exploring problems in depth.

## Figures and Tables

**Algorithm 1:**
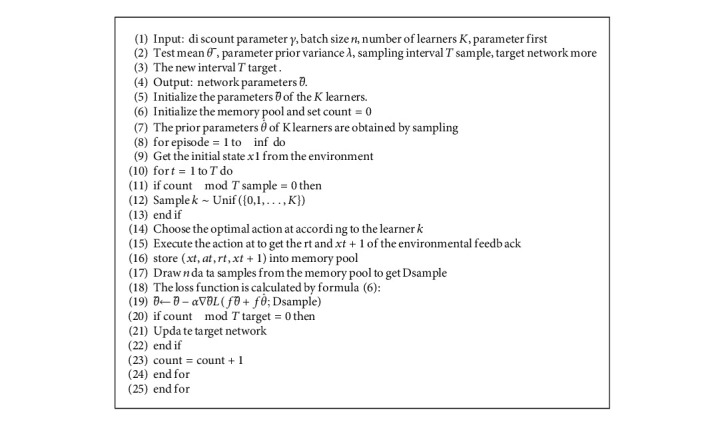
Bootstrapped DQN (BBDQN) algorithm.

**Table 1:** Hyperparameters.

Hyperparameters	Value
Discount parameters *γ*	0.99
Batch size *n*	128
Memory pool capacity	100
Number of learners *K*	10
Parameter prior mean *θˉ*	0
Parameter prior variance *λ*	10
Sampling interval *T*^sample^	20
Target network update interval *T*^target^	20

**Table 2:** The number of episodes required to find rewards.

*N*	*l*
5	560
10	792
15	1663
20	1828
25	2657
30	7697

**Table 3:** Cumulative regret of each algorithm in 10,000 episodes.

Variable settings		DQN	BDQN	BBDQN
*K* = 10	*N* = 10	9914.88	599.65	649.29
	*N* = 20	9922.02	9914.60	1590.03
	*N* = 30	9912.36	9910.96	5203.65

*N* = 20	*K* = 10	9922.02	9914.02	1590.03
	*K* = 20	9922.02	9912.13	1901.18
	*K* = 30	9922.02	1857.49	1784.71

## Data Availability

The data used to support the findings of this study are available from the corresponding author on request.
